# Supplementary material for: A Remote Sensing Approach for Assessing Daily Cumulative Evapotranspiration Integral in Wheat Genotype Screening for Drought Adaptation
Source: Plants (Basel). 2023 Nov 16;12(22):3871. doi: 10.3390/plants12223871 (PMC10675030; doi:10.3390/plants12223871)
Supplement: Supplementary file 1 [file plants-12-03871-s001.zip › plants-2685316-supplementary.pdf]

Supplementary Table S1. Pearson correlation coefficients and probabilities were computed for grain yield (GY, kg·ha-1) in relation to the following variables: leaf area index (LAI), cumulative actual evapotranspiration (ETa, mm), cumulative actual evapotranspiration during vegetative stages (ETa VEG, mm), cumulative actual evapotranspiration during grain filling (ETa GF, mm), and water productivity (WP, kg·m-3). The correlations were based on the means of each variety in two irrigation treatments and two years, resulting in a total sample size of 88.

	GY	
	r	p
ETa GF	**0.7745**	**<.0001**
ETa VEG	-0.0984	0.3619
WP	**0.9381**	**<.0001**
ETa	**0.7372**	**<.0001**
LAI_estimated	**0.5311**	**<.0001**

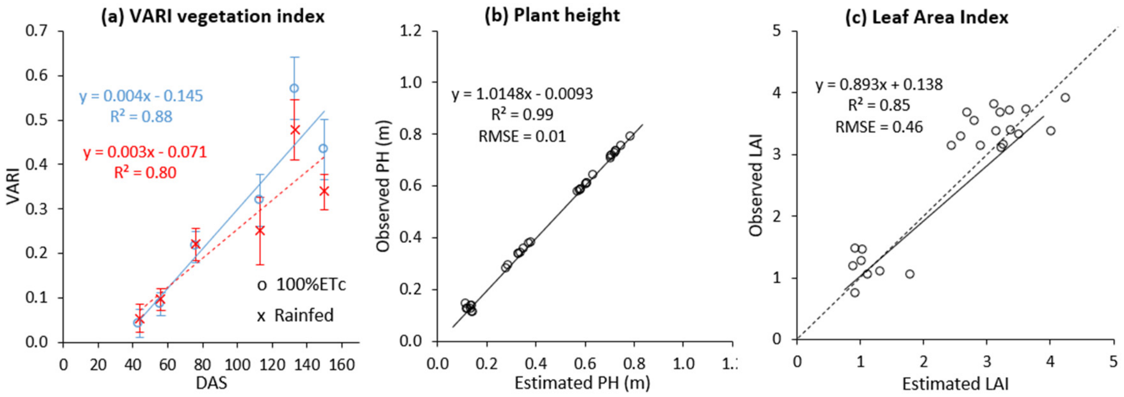

Supplementary Figure S1. (a) Seasonal evolution of VARI (Visible Atmospherically Resistant Index) vegetation index; (b) Relationship between observed and estimated plant height (PH); and (c) relationship between observed and estimated leaf area index (LAI) using a multiple regression analysis. DAS: Days After Sowing.

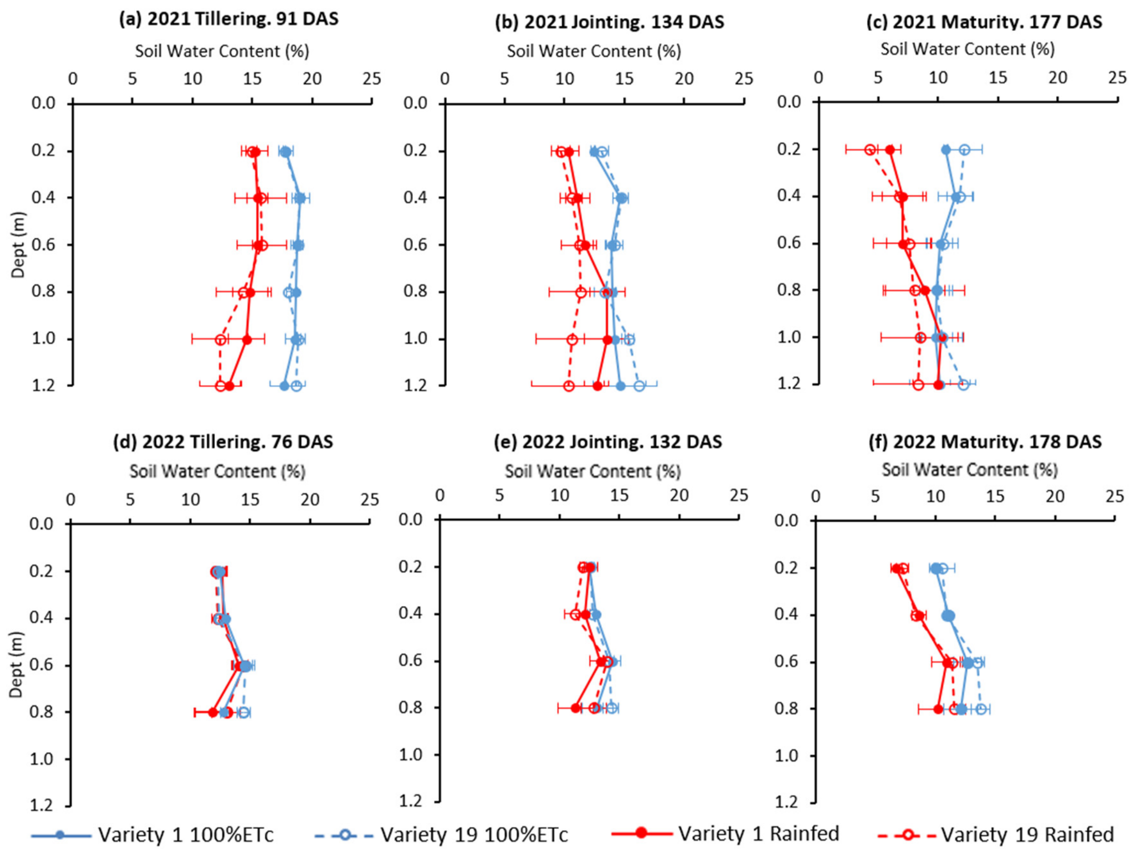

Supplementary Figure S2. Effect of water regime (100%ETc and rainfed) on soil water depletion along the soil profile (mean values ± standard error) on tillering, jointing and maturity crop stages of Variety 1 and Variety 19 wheat varieties in 2021 (a–c) and 2022 (d–f). Neutron probe-assessed mean values of 3 probes per treatment. DAS indicate Days After Sowing.
